# Methodological Validation and Inter-Laboratory Comparison of Microneutralization Assay for Detecting Anti-AAV9 Neutralizing Antibody in Human

**DOI:** 10.3390/v16101512

**Published:** 2024-09-24

**Authors:** Shuangqing Yu, Qian Zhao, Cengceng Zhang, Diyi Fu, Xueyang Zhu, Jianfang Zhou, Wenhao Ma, Zheyue Dong, Xiaoliang Zhai, Lijie Jiang, Xiaohong Han, Shuyang Zhang, Xiaobing Wu, Xiaoyan Dong

**Affiliations:** 1 Genecradle Therapeutics Inc., Beijing 100176, China; 2 Clinical Pharmacology Research Center, State Key Laboratory of Complex Severe and Rare Diseases, NMPA Key Laboratory for Clinical Research and Evaluation of Drug, Beijing Key Laboratory of Clinical PK and PD Investigation for Innovative Drugs, Peking Union Medical College Hospital, Chinese Academy of Medical Sciences & Peking Union Medical College, Beijing 100730, China; 3 Beijing FivePlus Gene Technology Co., Ltd., Beijing 102629, China; 4 Beijing Joinn Laboratory Co., Ltd., Beijing 100176, China; 5 State Key Laboratory of Complex Sever and Rare Diseases, Department of Cardiology, Peking Union Medical College Hospital, Chinese Academy of Medical Sciences & Peking Union Medical College, Beijing 100730, China

**Keywords:** adeno-associated virus, neutralizing antibody, microneutralization assay, sensitivity, specificity, precision, reproducibility, system suitability

## Abstract

Anti-AAV neutralizing Abs (NAbs) titer is usually measured by cell-based microneutralization (MN) assay and is crucial for patient screening in AAV-based gene therapy clinical trials. However, achieving uniform operation and comparable results among different laboratories remains challenging. Here, we established a standardized MN assay for anti-AAV9 NAbs in human sera or plasma and transferred the method to the other two research teams. Then, we validated its parameters and tested a set of eight human samples in blind across all laboratories. The end-point titer, defined by a transduction inhibition of 50% (IC_50_), was calculated using curve-fit modelling. A mouse neutralizing monoclonal antibody in human negative serum was used for system quality control (QC), requiring inter-assay titer variation of <4-fold difference or geometric coefficient of variation (%GCV) of <50%. The assay demonstrated a sensitivity of 54 ng/mL and no cross-reactivity to 20 μg/mL anti-AAV8 MoAb. The intra-assay and inter-assay variation for the low positive QC were 7–35% and 22–41%, respectively. The titers of the blind samples showed excellent reproducibility within and among laboratories, with a %GCV of 18–59% and 23–46%, respectively. This study provides a commonly transferrable MN assay for evaluating anti-AAV9 NAbs in humans, supporting its application in clinical trials.

## 1. Introduction

Adeno-associated viruses (AAVs) are promising vectors for gene therapy (GT). However, one major challenge for AAV-based GTs is the high prevalence of anti-AAV antibodies. Around 50–90% of the human population have antibodies against AAVs due to natural, nonpathogenic AAV infections. The prevalence of antibodies varies depending on AAV serotypes, geographic regions and age groups [1,2]. Serological investigations typically involve measuring either total AAV capsid-binding antibodies or neutralizing antibodies [3,4]. Total antibodies (TAbs), also known as binding antibodies, bind to viral antigens but do not definitively inhibit viral transduction. Neutralizing antibodies (NAbs), on the other hand, have the ability to inhibit viral transduction. In addition to NAbs, other matrix factors have been suggested to negatively impact cellular uptake and/or inhibit transgene expression. NAbs or inhibitory factors could block AAV transduction and potentially enhance the immunogenicity of AAV vectors [1,5,6,7,8]. Therefore, variations in pre-existing immunity to different AAV serotypes are crucial in selecting appropriate AAV serotypes for gene therapy. TAbs methods are usually ligand-binding assays that are relatively straightforward and robust to develop, while NAbs methods often involve more complex cell-based virus microneutralization (MN) assays. Current data is insufficient to establish a correlation between TAbs and NAbs levels. Recent reports suggest that antibodies binding to AAV without neutralizing activity may facilitate AAV transduction into hepatocytes in animal models, which implies that pre-existing NAb assays would have a more significant impact on AAV gene therapy. Regulatory guidelines recommend excluding patients with pre-existing antibody titers above a certain threshold in gene therapy, as the treatment efficacy may be reduced otherwise [9,10,11,12].

The cell-based MN test is a highly sensitive and specific assay by in vitro measurement on transduction inhibition (TI) of rAAVs with reporter genes, such as luciferase, GFP or β-galactosidase [9,13,14], into susceptible cells. The sensitivity, specificity, accuracy and cut-off of the methods depend on the characteristics of the NAbs assay, including the types of used cells, the multiplicity of infection (MOI) value, the use of some reagents to improve assay sensitivity, the selection of assay matrix and even the methods of result calculation [4,15,16,17]. It is challenging to develop a uniform NAbs assay to achieve consistency or standardization. Regulatory agencies such as the Food and Drug Administration (FDA), European Medicines Agency (EMA) and The National Medical Products Administration (NMPA) in China recommend that methods be initially deemed “fit for purpose” and subsequently validated for clinical studies to provide primary evidence of effectiveness for marketing application. However, there is no consensus in the industry on how to conduct methodological validation for cell-based AAV NAbs assays.

AAV9 vector, with broad tissue tropisms, is being widely used in GTs targeting neurological, muscular or cardiological diseases [18,19]. AAV9-SMN1 (named Zolgensma^®^) has been approved for intravenous injection for spinal muscular atrophy (SMA) patients while its inclusion/exclusion cut-off titer varied, including anti-AAV9 IgG < 1:50 or <1:400 or NAbs < 1:1 [8,9]. The increasing need to compare the levels of pre-existing AAV antibodies for enrollments of clinical trials, along with the AAV serological prevalence in populations and the data sharing on pharmacokinetics and efficacy of rAAVs, poses challenges for AAV-vector-based product enterprises, laboratories and licensing authorities.

Herein, we established an optimized anti-AAV9 NAbs MN assay with standardized critical materials, as well as positive and negative controls. The established methodology was transferred from the leading laboratory to the other two laboratories in Beijing. Validation of the MN assay was performed with reference to the 2021 NMPA immunogenicity guidance [9,10] in all laboratories. Then, its intra- and inter-laboratory variability and reproducibility were assessed using a set of human serum or plasma samples.

## 2. Materials and Methods

### 2.1. Cell Culture and rAAV-EGFP-2A-Gluc Viruses

Human embryonic kidney HEK293-C340 and HEK293-C018 cell lines, subcloned from ATCC CRL-1573 by Beijing Five Plus Gene Technology Company (Beijing, China), were maintained in DMEM supplemented with 10% FBS (D10) at 37 °C, 5% CO_2_. The master cell bank and working cell bank of HEK293-C340 were set, in which cells with a passage number of ≤50 are recommended.

The rAAV9 vectors encoding Gaussian luciferase under the control of the CMV enhancer/beta-actin (CA) promoter, namely rAAV9-EGFP-2A-Gluc, were produced using triple-plasmid transient transfection in suspension-cultured HEK293T cells into in a 200 mL single-use flask. These co-transfection plasmids include Rep2Cap9 plasmid, helper plasmid and packaging plasmid. Vector particles were released from harvested cells by adding detergent directly to the flask and purified as previously described [20]. The empty and full virus particles were separated by CsCl density gradient ultracentrifugation, and the percentage of empty capsids was maintained at <10%. The virus bulk was formulated in 0.001% Pluronic (F68) phosphate buffered solution (PBS) and stored at −80 °C. Genome titers, i.e., vector genome copies (vg) of AAV vectors, were determined by real-time PCR and with plasmid DNA as standards as reported previously [21]. Transducing titration of rAAV9-EGFP-2A-Gluc was performed on 20,000 HEK293-C340 cells, and the cells were then transfected with serial dilution of the virus ranging from 6.25 × 10^6^ to 8 × 10^8^ vg/well for 48 to 72 h. After incubation, the luciferase activity of transduced Gluc was measured by a luciferase assay system according to the manufacturer’s instruction. Briefly, 20 μL of supernatant from each well reacted with 50 μL of coelenterazine native substrate (Nanolight, Pinetop, AZ, USA) in a black 96-well plate at RT. The relative luciferase unit (RLU) was read by a GLOMAX 96 microplate luminometer (Promega, Madison, WI, USA). For the ongoing MN test, the titer of the virus stock at 2 × 10^8^ vg/well (MOI = 10^4^) must be able to generate a luminescence signal with a virus control (VC) to cell control (CC) ratio higher than 10 and R^2^ of a dose–response curve higher than 0.95.

### 2.2. Serum or Plasma Samples

Human blood samples, including serum or paired EDTA K_2_-anticoagulated plasma, were obtained from healthy adult donors with written informed consent after review by the corresponding ethical committee. The serum or plasma samples were aliquoted and stored at −30 °C. The animal serum was prepared by the bioanalysis group of Genecradle Therapeutics Inc. (Beijing, China) and the research protocol was reviewed by IRB.

### 2.3. Optimizing Key Variables for Anti-AAV9 MN Assay Development

The following factors, including selection of cell line, the number of cells, viral particle dose, incubation time and selection of sample matrix, were optimized for setting up the bioassay. The detection signal, the linear effect of the dose–response curve and the variation in titer were compared. Seventeen paired sera and EDTA K_2_-anticoagulated plasmas were assayed for the selection of the sample matrix.

### 2.4. AAV9 MN Assay Protocol

Serum or plasma samples for testing were pre-treated at 56 °C for 30 min. An amount of 50 µL of 2-fold serially diluted serum or plasma, starting with a dilution of 1:20, was incubated with 2 × 10^8^ vg of rAAV9-EGFP-2A-Gluc in 50 μL DMEM containing 0.1% BSA (Sigma, St. Louis, MO, USA) for 1 h at 37 °C. The assay was performed in triplicates, and then 20,000 HEK-293-C340 cells in 100 μL of D10 with 1 mM of sodium butyrate were added to 96-well plates. The plates were incubated at 37 °C under 5% CO_2_ for 48 to 72 h. After incubation, RLU was measured and wells with RLU CV% > 30% were excluded. Transduction inhibition (TI) was calculated based on the equation, [1 − (mean RLU_test_ − mean RLU_cc_)/(mean RLU_vc_ − mean RLU_cc_)] × 100%. The titer at which a serum inhibited 50% of the transduction (IC_50_) was calculated by 4-parameter logistics (PL) regression analysis using Prism software 8.0 (GraphPad Software, San Diego, CA, USA). An R^2^ value above 0.8 is required for IC_50_ titer calculation.

### 2.5. Quality Control

The negative sera confirmed by MN were pooled as the negative control (NC). The anti-AAV8, AAV9-specific monoclonal antibodies (MoAb) were generated by Genecradle Therapeutics Inc. (Beijing, China) using the hybridoma technique from specific serotype empty capsids-immunized Balb/C mice. The pooled negative sera or plasma spiked with anti-AAV9 MoAb was used as the positive control (PC). Low, middle and high concentration quality controls (LPC, MPC and HPC, respectively) were prepared at 200, 500 and 2000 ng/mL, respectively.

The NC, HPC and LPC were included in each assay round. The initial dilution of the PCs was 1:20, and 6–8 2-fold dilutions with D10 were made for each test run. The data were considered valid only when the parallel NC, LPC and HPC met the acceptance criteria.

### 2.6. Study Design, Method Transfer and Validation

The study includes 3 stages of activities. In stage I, the bioanalysis group of Genecradle Therapeutics Inc. established the standard operation procedure (SOP) and set NC, LPC and HPC for system suitability to ensure all critical reagents were qualified. The leading lab then transferred the methodology, including the SOP and key materials, to the other two laboratories and trained them in their own laboratories. Once routine cell culture and quality criteria were met, the assay parameters were validated by the laboratories themselves in stage II. The acceptance criteria for the parameters are listed in Table S1.

Method parameters including cut point, sensitivity, assay precision, specificity, selectivity, drug tolerance, robustness, stability and system suitability were evaluated. Analysis of different titration curves (NC, LPC, MPC and HPC) was performed in each lab within 3–6 days by 2 operators. An anti-AAV8 MoAb was used to test the assay specificity. Five individual hemolytic plasma (RBC vol/vol, 2%) or lipemia (triglyceride 11.3 mmol/L) samples were assayed for matrix interference in the assays.

In stage III, a set of 8 samples, including serum, plasma or commercial immunoglobulin (IVIG) labeled as S001–S008, were tested blindly by all laboratories. The detailed information is described in Table S2. The worksheets reporting the IC_50_ measurements for all assay, QC and analysis data were submitted to the leading laboratory for statistical analysis. The identity of each sample was revealed after detection for analysis. The operators were assigned code numbers 1–3.

### 2.7. Statistical Analysis

The data were analyzed using GraphPad Prism (version 8) and JMP statistical software (version 12). When the IC_50_ curve fitting failed and the TI% was <50% throughout all titrations, a titer of <1:20 was assigned a value of 1:10 for statistical purposes.

When the R^2^ of the curve was above 0.8, the titer at which a serum inhibited 50% of the transduction (IC_50_) was calculated by 4-parameter logistics (PL) regression analysis with Prism software 8.0.

Another end-point titer was based on the cut-off value, calculated using the following equation, X = ((mean RLU of VC wells) + (mean RLU of CC wells))/2. All values below or equal to X were considered positive for neutralization. The reciprocal of the last positive dilution was considered a cut-off titer.

The titer cut-point (TCP) of the assay is the response level that defines the sample as positive or negative [22] and was statistically designed to yield a 5% false positive rate (FPR). TCP was obtained from the negative samples after excluding samples that potentially contained pre-existing antibodies [23]. The titers were calculated as the geometric mean titer (GMT). Outliers were identified by the Shapiro–Wilk test. TCP was calculated by applying a 90% one-sided lower confidence interval for the 95th percentile of the negative control population.

Variation was expressed as the percentage geometric coefficient of variation (%GCV) and fold-change of the titers [24,25]. The GCV was calculated using the formula, GCV = $e^{\sqrt{\ln(1+{\left(\frac{SD}{\bar{X}}\right)}^{2})}}-1$.

To assess intra- and inter-laboratory reproducibility, a comparison was made of replicate assays; %GCV and fold variation were calculated for the tested 8 samples, yielding 5 sets of replicate titers. The difference between the GMT titers measured at different conditions was determined as the bias% calculated by the formula, (GMT_test_/GMT_basline_ − 1) × 100%.

## 3. Results

### 3.1. Anti-AAV9 MN Assay Establishment

**Figure 1:**
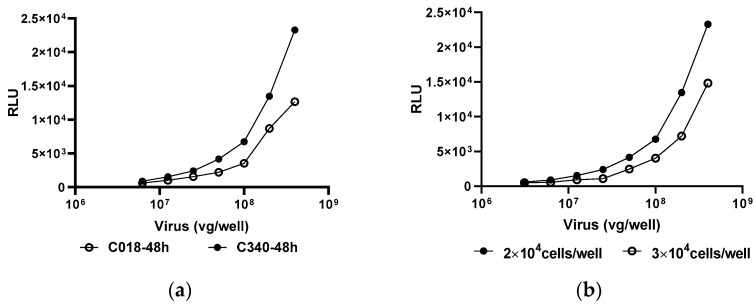

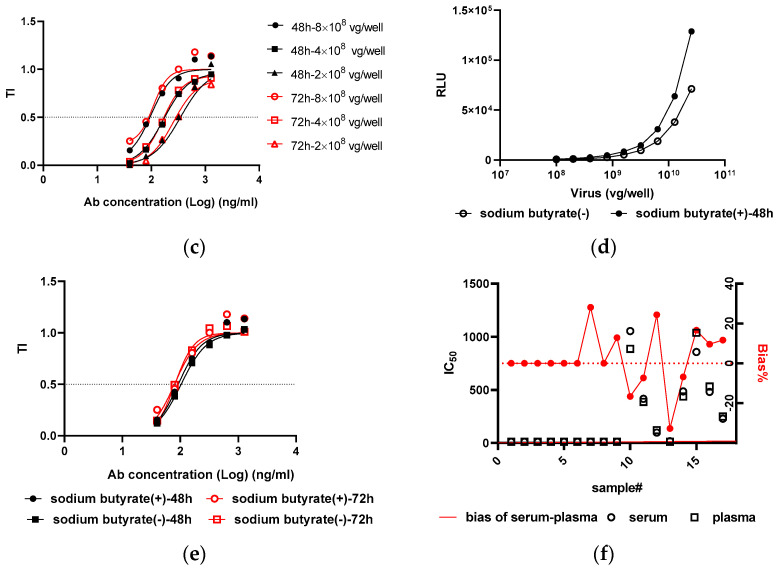
Anti-AAV9 MN assay establishment. The linear effects of each variable on the detection signal of Gluc expression or the transduction inhibition (TI) on AAV9-permissive cells for anti-AAV9 MN assay were tested. (**a**) the selection of a permissive cell line for anti-AAV9 MN assay. (**b**) the selection of cell numbers. (**c**) the TI obtained from 48 h- or 72 h-incubation at a virus dose of 2, 4 and 8 × 10^8^ vg/well (MOI = 1×, 2× and 4 × 10^4^, respectively). (**d**) the detection signal of Gluc expression in the presence of 1 mM sodium. (**e**) the TI obtained from an MN assay with the addition of 1 mM sodium. (**f**) the IC_50_ of antisera or EDTA K_2_-anticoagulated plasma using the AAV9 MN assay. HEK293-C340 or HEK293-C018 cells were infected with rAAV9-EGFP-2A-Gluc for 48 to 72 h and then the luminescence signal was detected. The transduction could be enhanced by the addition of 1 mM sodium butyrate. The anti-AAV9 NAbs titers of 17 paired sera and EDTA K_2_-anticoagulated plasma showed no significant difference and the bias% between them was within ±40%. The black dot denotes the TI of 50%. The red dot line in (**f**) denotes the bias of 0. The data was the mean of two experiments. relative luciferase units (RLU); 50% of the transduction (IC_50_).

As demonstrated in Figure 1, the linear effect of each variable on the detection signal of the anti-AAV9 MN assay was tested. Initially, we used the serum from naive and AAV9-treated mice to explore the minimal required dilution (MRD). We found that the MRD for the serum sample was 1:20, as a consistently low signal was observed across all dilutions <1:20, and poor cell growth was noted in the presence of a high concentration of serum. Similar results were also found with human serum samples. Generally, the sensitivity of an assay was dependent on the detection signals of the test, which implies the fewer viruses used, the greater the sensitivity in the MN assay. Therefore, we aimed to obtain signals with an appropriate virus dose. We found that detection signals were related to the selection of cell line (Figure 1a), cell numbers (Figure 1b), AAV dose and incubation time (Figure 1c). As shown in Figure 1c, the IC_50_ value is sensitive to the virus dose, with a higher titer obtained in the presence of a lower virus dose. Although the detection signals increased as the Gluc accumulated in the supernatants with time, the IC_50_ measured at 48 h or 72 h showed no difference.

Furthermore, the detection signals could be enhanced by the addition of 1 mmol/L sodium butyrate (Figure 1d,e). Finally, 2 × 10^4^ HEK293-C340 cells were transfected with 2 × 10^8^ vg/well rAAV9-EGFP-2A-Gluc for 48 to 72 h, generating a mean luminescence signal for VC to CC that was above 10.

Then, we compared the anti-AAV9 NAbs titers of 17 paired sera and EDTA K_2_-anticoagulated plasma (Figure 1f), the bias % between them was within ±40%. We also assessed the intra-assay variability by repeatedly measuring the NAbs titer of PC (mouse anti-AAV9 MoAb at a concentration of 100 ng/mL). The coefficient of variation (CV) of its IC_50_ value in six independent experiments was 10%, indicating low variability (Table 1).

### 3.2. Key Parameters Validation

To ensure that it could be applied in clinical practice, we complied with the NMPA guidance and validated the assay parameters, including TCP, precision, sensitivity, drug tolerance and hook effect, specificity, robustness, stability and system suitability. The overall parameters validated by each laboratory are listed in Table 2; the raw data in Tables S3–S15 are included in the Supplementary Document. The validation details are introduced as follows.

#### 3.2.1. TCP Assay

Twenty-six to fifty serum samples from healthy donors were assayed six times in triplicates by two analysts over three days in six experiments using the MN assay. Values from the individual runs are shown in Table S4. The distribution of IC_50_ values was utilized to identify analytical outliers using the Tukey box-plot outlier criteria. After excluding outliers, the distribution of the remaining log-10 transformed normalized dataset and the absolute Skewness value were evaluated. Since the Skewness was >1, the TCP was set as the 95th percentile value for the entire dataset. The TCP was set at 1:21 in Laboratory 1 and 1:15 in Laboratory 3.

#### 3.2.2. Assay Precision

Inter- and intra-assay precision were assessed based on the IC_50_ variation of six sets of NC, LPC, MPC and HPC tested in MN assay performed six times in triplicates by two analysts over two days. Precision data from three laboratories are summarized in Table 2, and detailed information is provided in Tables S5–S7. The acceptance criteria of the target were <4-fold difference or %GCV values < 50%. The results demonstrated that the intra-assay precision met the acceptance criteria except in one of six runs in Lab1, where HPC replicates had a %GCV of 52%, but the fold-change was <4-fold.

#### 3.2.3. Sensitivity and Hook Effect

The NAb assay sensitivity was calculated from six individual runs with PC (anti-AAV9 MoAbs stock at eight concentrations from 3200 ng/mL to 25 ng/mL). Then, a serial half dilution was performed on these stocks and their IC_50_ values were assayed using the MN assay. The sensitivity of the assay was calculated based on the mean of the back-calculated concentrations of the titration–TI curves, which were 54, 45 and 15 ng/mL in the three laboratories, respectively. No hook effect was observed, as demonstrated by the fact that the calculated concentrations did not decrease with increasing concentrations in the range of 25–10, 000 ng/mL PC (Table S8).

#### 3.2.4. Drug Tolerance

**Figure 2:**
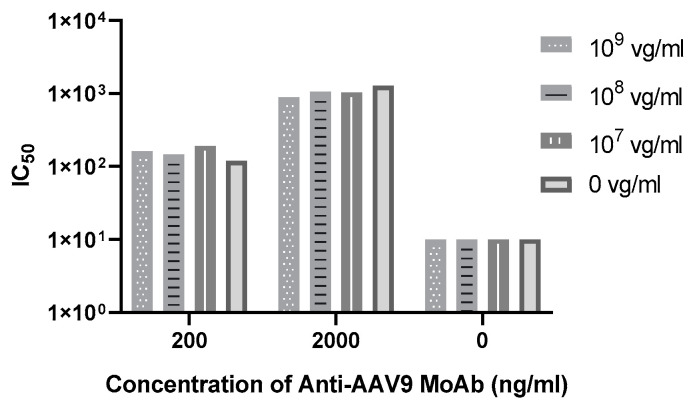
Drug tolerance of the MN assay. The IC_50_ of NC and PCs at concentrations of 200 and 2000 ng/mL in the presence of rAAV9 drug G301 at final concentrations of 10^9^, 10^8^ and 10^7^ vg/mL. The IC_50_ titer showed no difference in the presence of rAAV9, and raw data was attached in Table S9.

Since approximately 10^8^ vg/mL of DNA vector copies could be detected in blood on Day 7 after infusing 1.2 × 10^14^ vg/kg of GC301, a rAAV9-coGAA vector for the treatment of Pompe disease, we measured the IC_50_ of NC and PCs at concentrations of 200 and 2000 ng/mL in presence of G301 at a dose of 10^9^, 10^8^ and 10^7^ vg/mL. Both NC and spiked PCs showed no difference compared to those without GC301. The finding indicated that the assay is tolerant of the rAAV9 drug up to 10^9^ vg/mL (Figure 2 and Table S9).

#### 3.2.5. Specificity

We then used anti-AAV8 MoAbs to assess the specificity of the MN assay. The LPC or NC was incubated with 2 μg/mL or 20 μg/mL of anti-AAV8 MoAb. A comparison of their IC_50_ values showed no significant difference (Table S10), indicating the high specificity of the MN assay.

#### 3.2.6. Robustness and Stability

The robustness of the assay was examined by testing the PCs under different assay conditions. Three factors were investigated on different days: pre-incubation time for the sample and virus, passage number and total incubation time of the cells. A comparison of the IC_50_ titers and their variations showed that all values were acceptable and only small variations were observed (Tables S12 and S13), suggesting that the assay robustness is excellent. The HEK293-C340 cells for testing were maintained for up to 50 passages, the incubation time for serum and virus was approximately 1–2 h and the total incubation time was around 2 days.

The short- and long-term stability, as well as the storage condition, freeze/thaw cycles and storage duration of the PCs were tested. The titer bias was within ±40%, and no significant decrease was observed in the titers of LPC and HPC at 25 °C for 24 h, −30 °C for 9 months, −70 °C for 5 months and freeze/thaw cycles within six times (Table S14).

#### 3.2.7. System Suitability

We obtained the IC_50_ of PCs under actual use and tested their consistency to evaluate system suitability. The GMTs of LPC and HPC were obtained from all the tests using the MN assay. The range for GMT was determined based on the change between the lower and upper limits that were within 4-fold. The ranges for LPC across three laboratories were 1:34–1:135, 1:35–1:139 and 1:74–1:295, respectively. For HPC, the ranges were 1:383–1:1532, 1:283–1:1132 and 1:621–1:2486, respectively (Table S15).

#### 3.2.8. Assay Repeatability

**Figure 3:**
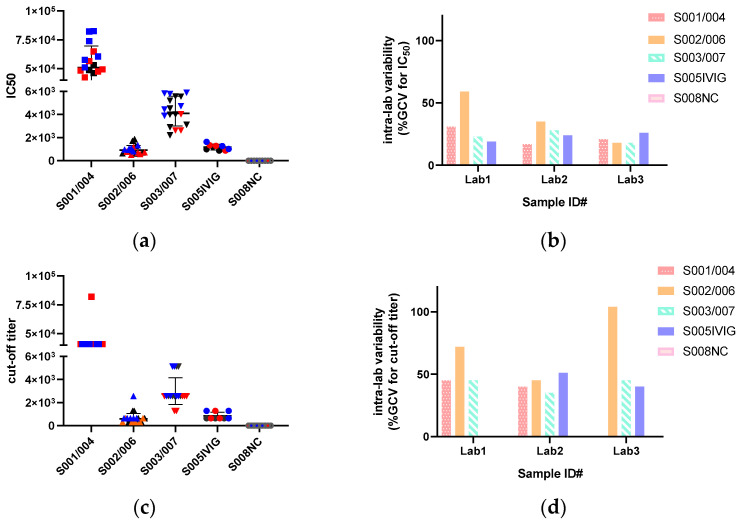

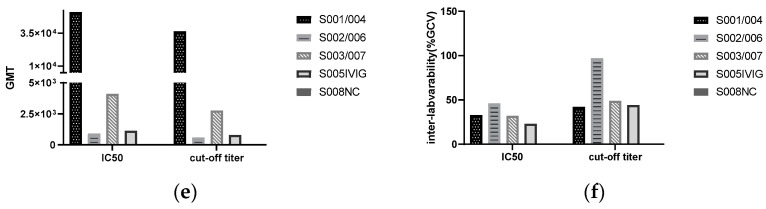
The anti-AAV9 NAbs titer, intra-lab variability and inter-lab variability of the blind human samples. (**a**) the IC_50_ of the sample set, including samples 001, 002, 003 with their identical samples 004, 006 and 007, commercial immunoglobulin (IVIG, S005) and negative control sera pool (NC, S008) assayed by 3 laboratories. (**b**) the intra-variability on IC_50_ of three paired samples, IVIG and NC of each laboratory. (**c**) the endpoint of the sample set expressed as the cut-off titer. (**d**) the intra-lab variability of the sample set read as the cut-off titer. (**e**) the comparison on GMT of the sample set read by IC_50_ and the cut-off titer. (**f**) Inter-lab variability for IC_50_ and the cut-off titer of 3 paired samples, IVIG and NC sample. These samples were run blind in each assay by all laboratories. In (**a**,**c**), different colors denote different laboratories: black for Laboratory 1, red for Laboratory 2, and blue for Laboratory 3. The IC_50_ was calculated by 4PL regression analysis. The cut-off titer was based on the value, calculated as the ((mean RLU of VC wells) + (mean RLU of CC wells))/2. All values below or equal to this cut-off were considered positive for neutralization. The reciprocal of the last serum dilution for positive was the neutralization antibody titer, namely the cut-off titer. Information on the sample set is attached in Table S2. Raw data was attached in Tables S16 and S17.

We used a set of human serum or plasma samples to evaluate their repeatability (Figure 3a,b and Table S16). We first evaluated the reproducibility within laboratories. The NC titer (S008) was consistently equal to 1:10 across experiments in all laboratories. When comparing the IC_50_ of samples 001, 002 and 003 with their identical samples 004, 006 and 007 within the discrete test, respectively, only one laboratory (Laboratory 1) showed an IC_50_ titer for S002 and 006 that varied between 2-fold and 4-fold, with the %GCV of repeated tests being 52% and 53%, respectively. Intra-laboratory reproducibility was also assayed by comparing the inter-assay variation on the replicate titers of each sample within each laboratory. Based on three or six replicates for S001/004, 002/006 and S003/007, the %GCV for IC_50_ of the test sera ranged from 18% to 59%. Only one sample had a %GCV of 59%. In most laboratories, the reproducibility was good, with %GCV < 50% and fold-change < 4-fold.

Initially, we designed IVIG (S005) as an internal standard and intended to compare both absolute value and normalized data to IVIG. The IC_50_ for IVIG was stable across laboratories, with a GMT between 1:1026 and 1:1185. The intra-lab and inter-lab variability were 19–26% and 23%, respectively (Tables S16 and S17). We found that the reproducibility of the anti-AAV9 NAbs titer, regardless of whether assayed in the same or different labs without any data transformation, demonstrated high concordance.

We then assessed the reproducibility of the titers of tested sera between laboratories. For all sera, the inter-laboratory agreement was excellent, with variations reaching <50% (Figure 3f and Table S17).

### 3.3. IC_50_ Are Less Variable than Cut-Off Titers

In our MN assay, the end-point titer with a TI of 50% (IC_50_) was calculated using 4PL curve fitting on the titration–TI curve. This analysis required that both the AAV dose and the Ab titer–TI curve were within the linear region for IC_50_ calculation. As demonstrated in Figure 3a,c,e, the GMTs counted by IC_50_ titer and cut-off titer varied. Additionally, the intra- and inter-lab variability between IC_50_ and cut-off titer was 18–59% vs. 35–104% and 23–46% vs. 42–97%, respectively (Figure 3f), indicating that IC_50_ was less variable than cut-off titers.

## 4. Discussion

The quantification of anti-AAV Abs is important for patient enrollment in clinical trials. Typically, measurements on the TAbs or NAbs are used, but their performance requires a well-equipped laboratory and well-trained staff. Moreover, the transferability of methods or comparability of results among different laboratories has not been reported previously. This study reported a semi-quantitative HEK293-C340-based microneutralization assay for detecting anti-AAV9 NAbs. We present its transferability and method parameters and confirm the robustness of the MN assay. Furthermore, a set of eight human sera or plasma at different time points post administration of rAAV9 vectors (GC101 and GC301), IVIG and negative control serum were blindly tested and analyzed by participants. The data revealed that this MN assay can be applied in discrete laboratories with good intra- and inter-laboratory variability, in which the quality of key materials and modeling of the IC_50_ endpoint is critical.

The validations of the cell-based neutralizing assay by measuring TI have been documented for AAV5 [15], AAV6 [26] and AAVrh10 [27], with different assay protocols. For AAV5, the assay used HEK-293T/17 and set the cut point to transduction < 44.9%, with an MRD of 1:20 and an FPR of 1%. For AAV6, the U-87 MG human glioma cell line was used, with an MRD of 1:10 followed by four serial 2.5-fold dilutions. A clinical cut-off was designed statistically to yield an approximate 0.1% FPR using the normalized response (NR) compared to the negative control serum, with a cut-off of 0.34. For AAVrh10, HEK-293-2V6.11 was used, with an MRD of 1:2 followed by four serial 2–4-fold dilutions; a 1% FPR was set and the normalized NR was 0. 587. The cut-off values for different AAV serotypes in current GT trials also vary [17]. The anti-AAV9 NAbs assay has been performed in tests for sera samples from human or rhesus macaques while no method validation has been reported until now. For HEK293, HEK293T, HEK293H and Hela cells with a MOI of 2.5 × 10^4^ or 3.3 × 10^5^, the reporter gene green fluorescent protein or firefly luciferase were used in those tests [28,29,30,31,32].

There is lower transduction in AAV9 in vitro compared with other AAV serotypes such as AAV2, AAV5 and AAV6. To enhance the sensitivity of the MN assay, we have tried the following approaches: (1) the use of HEK293-C340 cells, a highly permissive subclone for AAV transduction; (2) the use of Gaussia luciferase (Gluc) reporter gene for its secreted transgene product, whose levels in supernatants are readily quantified by addition of coelenterazine. A study has compared the sensitivity of clormic and chemiluminescent signals for detecting anti-AAV antibodies, which found that chemiluminescent signals are more sensitive [33]; (3) the addition of sodium butyrate in the MN test enhances the transduction. Furthermore, an endpoint for the IC_50_ titer was introduced, and an S-shaped titration curve that contains a linear range followed by a plateau was obtained through curve-fit modeling. The modeled IC_50_ is less variable and is easier to calculate than the titer denoted as the reciprocal of the last dilution, causing a 50% decrease in RLU.

Since the rAAV9-EGFP-2A-Gluc used in the assay is an experimental material of critical importance, optimization of the amount of used virus and the linear range of viral titration needs to be guaranteed. Additionally, a specific anti-AAV9 MoAb was used as a quality control. Its IC_50_ is around 7 ng/mL. In the MN assay, 200 ng/mL and 2000 ng/mL were selected as the LPC and HPC, respectively. The sensitivity value for the QC in the assay is 15–54 ng/mL.

To test the feasibility of the bioanalysis performed by an inexperienced contract research organization (CRO) or clinical center, the leading lab provided the key materials including permissive cells, rAAV9-EGFP-2A-Gluc and QC reagents to the CRO and the hospital lab. Guided by the optimized MN SOP, the assay was established in both labs within 2 weeks. The MRD was 1:20, and TCP was statistically determined based on 5% FRP in healthy adults due to the limited availability of target pediatric patients. The method parameters were evaluated according to NMPA immunogenicity guidance. The assay parameters across the laboratories demonstrated good consistency, thus supporting assay robustness. We then mimicked the actual assay using a set of human samples. Initially, we designed IVIG as an internal standard to compare the absolute value and normalized data to IVIG. We found that the reproducibility of the anti-AAV9 NAbs titer, regardless of whether assayed by the same or different labs, is good without any data transformation.

We combined the MN assay with a passive immunized mice model and applied multiple parameters to evaluate the impact of different levels of pre-existing NAbs, which have helped us determine the exclusion criteria in clinical trials for AAV9-based Pompe disease gene therapy, as reported previously [6]. We have applied the MN assay in AAV9-based GT clinical trials, e.g., clinical trials of AAV9 expressing human acid alpha-glucosidase (GAA) gene therapy for patients with Pompe Disease (www.ClinicalTrials.gov ID NCT05567627, NCT05793307 and NCT06391736), and gene therapy drug GC101 in the treatment of spinal muscular atrophy (SMA) patients (www.ClinicalTrials.gov ID NCT05901987, NCT05824169 and NCT06421831). Moreover, it has been applied in a serological epidemiological investigation of anti-AAV9 NAbs in China and the data is currently being analyzed.

## 5. Conclusions

In summary, the MN assay for anti-AAV NAbs described herein is suitable for human serum and plasma samples, and is transferrable to a CRO or a clinical center that has not previously performed the assay. Notably, its accuracy was achieved by strictly implementing SOPs, standardizing key materials, QC criteria and modeling the IC_50_ endpoint. The validation and application data support its utilization in clinical trials. The assay may be further developed as a companion diagnostic test or criterion for treatment once the AAV9-mediated gene therapy is approved.

## Figures and Tables

**Table 1:** IC_50_ of a mouse neutralizing anti-AAV9 MoAb using the optimized MN assay.

	RA1	RA2	RA3	RA4	RA5	RA6	CV
IC_50_ (ng/mL)	7.35	7.8	6.29	6.45	7.69	6.29	10%
95%CI of IC_50_	6.47~8.35	6.07~10.76	5.82~6.81	4.22~10.55	5.82~10.29	5.82~6.81	-
R^2^	0.99	0.97	0.99	0.94	0.97	0.99	-

RA, round of analysis.

**Table 2:** The assay parameters in the study.

Parameters	Lab 1	Lab 2	Lab 3
TCP determination	1:21	-	1:15
Intra-assay and inter-assay precision (%GCV, fold change in titer)			
NC	0–58%, 1–2; 39%, 3	0–38%, 1–2; 19%, 2	11–31%, 1–3; 19%, 2
LPC	7–35%, 1–2; 37%, 3	10–35%, 1–2; 41%, 3	8–26%, 1–2; 22%, 2
MPC	9–36%, 1–2; 30%, 3	4–39%, 1–2; 28%, 2	11–24%, 1–2; 29%, 2
HPC	41–52%, 1–3; 33%, 4	6–40%, 1–2; 39%, 3	8–19%, 1–2; 18%, 2
Sensitivity (ng/mL) and Hook effect	54; 25~3200 ng/mL PC No hook effect	45	15; 100~10,000 ng/mL PC No hook effect
drug tolerance	-	-	Tolerant to 10^9^ vg/mL
Specificity			
	No cross-reactivity to 20 μg/mL anti-AAV8 MoAb	-	No cross-reactivity to 20 μg/mL anti-AAV8 MoAb
Selectivity		-	
hemolysis interference	100% pass at 0, 200 ng/mL PC	-	100% pass at 0, 200 ng/mL PC
lipemia interference	100% pass at 0, 200 ng/mL PC	-	100% pass at 0, 200 ng/mL PC
System suitability (%GCV, fold change in titer; GMT range)			
LPC	37%, 3; 34–135	41%, 3; 35–139	44%, 2; 74–295
HPC	33%, 3; 383–1532	39%, 3; 283–1132	16%, 2; 621–2486
Robustness and Stability			
	Pre-incubation time: 1 h ± 10 min; HEK-293T-C340: P17–50; incubation time: 48 ± 4 h LPC is stable at 4, 25, −30, −80 °C for short- and long-term storage and six times freeze/thawing	-	Pre-incubation time: 1–2 h; HEK-293T-C340: P21–24; incubation time: 44–64 h LPC is stable at 4, 25, −30, −80 °C for short- and long-term storage and 6 times freeze/thawing

- denotes not tested. GCV, geometric coefficient of variation; TCP, titer cut point; NC, Negative control; LPC, MPC and HPC are low, middle and high concentration quality controls, respectively. vg, vector genome copies.

## Data Availability

Materials and protocols will be distributed to qualified scientific researchers for noncommercial, academic purposes. The rAAV9-coGAA vector (GC301) and the vector sequence are part of an ongoing development program, and they will not be shared.

## Supplementary Materials

The following supporting information can be downloaded at: https://www.mdpi.com/article/10.3390/v16101512/s1, Table S1. Criteria for analytical validation. Table S2. Study sample set. Table S3. List of Abbreviations. Table S4. Raw data for TCP of the anti-AAV9 MN assay conducted by Laboratory 1 and 3. Table S5. Precision of the anti-AAV9 MN assay conducted by Laboratory 1. Table S6. Precision of the anti-AAV9 MN assay conducted by Laboratory 2. Table S7. Precision of the anti-AAV9 MN assay conducted by Laboratory 3. Table S8. Sensitivity and hook effects of the anti-AAV9 MN assay conducted by Laboratory 1, 2 and 3. Table S9. Drug tolerance of the anti-AAV9 MN assay conducted by Laboratory 2. Table S10. Specificity of the anti-AAV9 MN assay conducted by Laboratory 1 and 3. Table S11. Selectivity of the anti-AAV9 MN assay conducted by Laboratory 1 and 3. Table S12. Robustness of the anti-AAV9 MN assay conducted by Laboratory 1. Table S13. Robustness of the anti-AAV9 MN assay conducted by Laboratory 3. Table S14. Stability of the anti-AAV9 MN assay conducted by Laboratory 1 and 3. Table S15. System suitability of the anti-AAV9 MN assay conducted by Laboratory 1, 2 and 3. Table S16. Reproducibility of anti-AAV9 NAbs of eight human samples within laboratories. Table S17. Inter-lab and intra-lab variability of anti-AAV9 NAbs of eight human samples among three laboratories.
