# Supplementary material for: Methodological Validation and Inter-Laboratory Comparison of Microneutralization Assay for Detecting Anti-AAV9 Neutralizing Antibody in Human
Source: Viruses. 2024 Sep 24;16(10):1512. doi: 10.3390/v16101512 (PMC11512302; doi:10.3390/v16101512)
Supplement: Supplementary file 1 [file viruses-16-01512-s001.zip › Table S1 criteria for analytical validation.pdf]

Table S1. criteria for analytical validation

Parameters	Target acceptance criteria
TCP determination	the titer of negative population statistically to yield a 5% false positive rate
Sensitivity	The mean of the lowest concentration for PC back-calculated concentration at the MN assay. Less than or equal to 100 ng/mL
Intra-assay and inter-assay precision	NC:66.7% samples in titers with a %GCV of <50.0%
	LPC & HPC:Intra- and inter-assay %GCV in titers must be <50.0% or <4-fold difference
Specificity	20000 ng/ml anti-AAV8 MoAb that does not interfere with the LPC on AAV9 transduction
Selectivity	>80% LPC spiked in hemolytic or lipemia serum
System suitability	LPC&HPC: bias %<±40%
End-point	IC_{50} was calculated by 4-parameter logistics regression analysis. The R^2 of the curve is above 0.8
