# Supplementary material for: Methodological Validation and Inter-Laboratory Comparison of Microneutralization Assay for Detecting Anti-AAV9 Neutralizing Antibody in Human
Source: Viruses. 2024 Sep 24;16(10):1512. doi: 10.3390/v16101512 (PMC11512302; doi:10.3390/v16101512)
Supplement: Supplementary file 1 [file viruses-16-01512-s001.zip › Table S10 specificity.pdf]

Table S10 specificity

data on method validation in each laboratory

Lab 1				
the concetration of anti-AAV8 in the tested samples(ng/mL)	Tested sample	IC_{50}	mean of IC_{50}	R^2
20000	SPC-LPC-1	77	65	0.99
	SPC-LPC-1	49		0.99
	SPC-LPC-1	74		0.97
	SPC-NC-1	22	12	0.92
	SPC-NC-1	10		0.47
	SPC-NC-1	8		0.89
2000	SPC-LPC-1	81	74	0.95
	SPC-LPC-1	67		0.98
	SPC-LPC-1	74		0.97
	SPC-NC-1	19	8	0.94
	SPC-NC-1	10		0.22
	SPC-NC-1	3		0.87

Lab 3				
the concetration of anti-AAV8 in the tested samples(ng/mL)	Tested sample	IC_{50}	mean of IC_{50}	R^2
20000	SPC-LPC-1	113	191	0.98
	SPC-LPC-2	159		0.96
	SPC-LPC-3	301		0.89
	SPC-NC-1	10	14	NA
	SPC-NC-2	10		NA
	SPC-NC-3	21		0.83
2000	SPC-LPC-1	108	151	0.95
	SPC-LPC-2	199		0.97
	SPC-LPC-3	146		0.98
	SPC-NC-1	10	10	NA
	SPC-NC-2	10		NA
	SPC-NC-3	10		NA
