# Supplementary material for: Methodological Validation and Inter-Laboratory Comparison of Microneutralization Assay for Detecting Anti-AAV9 Neutralizing Antibody in Human
Source: Viruses. 2024 Sep 24;16(10):1512. doi: 10.3390/v16101512 (PMC11512302; doi:10.3390/v16101512)
Supplement: Supplementary file 1 [file viruses-16-01512-s001.zip › Table S11 selectivity.pdf]

Table S11 selectivity

data on method validation in each laboratory

Lab 1				percentage of samples spiked
concentration of PC （ng/mL）	sampleID	IC_{50}	R^2	
200	LPC-SEL01	127	0.91	100%
	LPC-SEL02	82	0.94	
	LPC-SEL03	80	0.98	
	LPC-SEL04	128	0.96	
	LPC-SEL05	107	0.95	
	LPC-SEL06	86	0.96	
	LPC-SEL07	70	0.92	
	LPC-SEL08	67	0.98	
	LPC-SEL09	106	0.98	
	LPC-SEL10	128	0.99	
0	NC-SEL01	10	NR	
	NC-SEL02	10	-3.03	
	NC-SEL03	11	0.66	
	NC-SEL04	10	0.04	
	NC-SEL05	10	0.27	
	NC-SEL06	10	-3.87	
	NC-SEL07	10	-4.55	
	NC-SEL08	10	-3.85	
	NC-SEL09	10	-0.34	
	NC-SEL10	10	-4.36	100%
200	LPC-Lip-1	129	0.98	100%
	LPC-Lip-2	102	0.98	
	LPC-Lip-3	124	0.99	
	LPC-Lip-4	141	0.95	
	LPC-Lip-5	141	0.92	
	LPC-Hae-1	94	0.99	100%
	LPC-Hae-2	75	0.91	
	LPC-Hae-3	87	0.92	
	LPC-Hae-4	84	0.91	
	LPC-Hae-5	111	0.99	
0	NC-Lip-1	10	-11.08	
	NC-Lip-2	10	-35.16	
	NC-Lip-3	10	-43.43	
	NC-Lip-4	10	-2.47	
	NC-Lip-5	10	-4.26	
	NC-Hae-1	10	-0.16	
	NC-Hae-2	10	-0.79	
	NC-Hae-3	10	-0.74	
	NC-Hae-4	10	-0.75	
	NC-Hae-5	10	0.47	100%

Lab 2				percentage of samples spiked
concentration of PC （ng/mL）	sample ID	IC_{50}	R^2	
	LPC-SEL01	207	0.85	
	LPC-SEL02	161	0.94	
	LPC-SEL03	247	0.88	
	LPC-SEL04	204	0.90	

200	LPC-SEL05	205	0.90		
	LPC-SEL06	138	0.91		
	LPC-SEL07	213	0.95		
	LPC-SEL08	165	0.94		
	LPC-SEL09	345	0.91		
	LPC-SEL10	155	0.95		100%
0	NC-SEL01	10	NA		
	NC-SEL02	10	NA		
	NC-SEL03	12	0.92		
	NC-SEL04	10	NA		
	NC-SEL05	3	0.97		
	NC-SEL06	10	NA		
	NC-SEL07	10	NA		
	NC-SEL08	9	0.81		
	NC-SEL09	5	0.81		
	NC-SEL10	10	NA		100%
200	LPC-Lip-1	182	0.89		
	LPC-Lip-2	159	0.91		
	LPC-Lip-3	174	0.97		
	LPC-Lip-4	223	0.88		
	LPC-Lip-5	234	0.96		100%
	LPC-Hae-1	208	0.90		
	LPC-Hae-2	122	0.94		
	LPC-Hae-3	196	0.94		
	LPC-Hae-4	270	0.94		
	LPC-Hae-5	223	0.94		100%
0	NC-Lip-1	10	NA		
	NC-Lip-2	10	NA		
	NC-Lip-3	10	NA		
	NC-Lip-4	10	NA		
	NC-Lip-5	10	NA		
	NC-Hae-1	10	NA		
	NC-Hae-2	14	0.81		
	NC-Hae-3	10	NA		
	NC-Hae-4	10	NA		
	NC-Hae-5	10	NA		100%
