# Supplementary material for: Methodological Validation and Inter-Laboratory Comparison of Microneutralization Assay for Detecting Anti-AAV9 Neutralizing Antibody in Human
Source: Viruses. 2024 Sep 24;16(10):1512. doi: 10.3390/v16101512 (PMC11512302; doi:10.3390/v16101512)
Supplement: Supplementary file 1 [file viruses-16-01512-s001.zip › Table S12 robustness lab1.pdf]

Table S12 robustness lab1

data on method validation in each laboratory

Lab 1

AR ID	pre-incubation time for	passage of HEK	incubation time	QC	IC50	QC	IC50	QC	IC50	QC	IC50
1	70 min	P50	52h	NC	10	LPC	67	MPC	242	HPC	885
					10		90		303		977
					NR		NR		NR		NR
					NR		NR		NR		NR
					10		74		235		1031
					10		67		218		1032
2	63 min	P17	4h，14m	NC	25	LPC	96	MPC	198	HPC	762
					4		90		190		1023
					10		94		182		876
					11		82		150		757
					19		78		159		750
					15		82		147		813
3	50 min	P18	44h	NC	10	LPC	89	MPC	273	HPC	1089
					15		66		200		807
					2		66		196		1164
					10		73		176		766
					10		59		140		907
					NR		NR		NR		NR

analysis data

QC	GMT	QC	GMT	QC	GMT	QC	GMT
NC	10	LPC	74	MPC	248	HPC	979
NC	12	LPC	87	MPC	170	HPC	825
NC	8	LPC	70	MPC	192	HPC	934
