# Supplementary material for: Methodological Validation and Inter-Laboratory Comparison of Microneutralization Assay for Detecting Anti-AAV9 Neutralizing Antibody in Human
Source: Viruses. 2024 Sep 24;16(10):1512. doi: 10.3390/v16101512 (PMC11512302; doi:10.3390/v16101512)
Supplement: Supplementary file 1 [file viruses-16-01512-s001.zip › Table S13 robustness lab3.pdf]

Table S13 robustness lab3

data on method validation in each laboratory

Lab 3

AR ID	pre-incubation time for	passage of HEK29	incubation time	QC	IC50					
1	2h	P24	44h	NC	10	7.8	7.1	9.5	10	10
2			48h		10	5.6	10	10	10	10
3			64h		6.5	10	10	10	10	10
4	1h	P21	50h		10	3.9	10	10	10	10
5					7.5	10	10	10	10	10
6					10	10	10	10	5.4	10
1	2h	P24	44h	LPC	112.2	188.8	129.7	157.5	137.2	155.2
2			48h		200.8	173	238.8	231.4	219.6	189
3			64h		136.3	164.1	163.8	107.4	213.1	195.1
4	1h	P21	50h		156.7	110.9	155.8	129.5	144.8	174.1
5					153.9	136.8	161.6	220.2	153.2	175.7
6					190.5	164.3	204.3	202.9	198.8	187.8
1	2h	P24	44h	MPC	369.5	638	534.9	505.4	464.6	439.6
2			48h		368.8	490.6	593.7	551.2	635.6	644.5
3			64h		523.7	514.6	466.1	352.1	491.1	656.6
4	1h	P21	50h		332.8	360.9	516	321.6	434.2	303.6
5					267.8	304.2	413.6	469.2	342.7	360.7
6					623.5	609.3	766.8	607.1	569.9	624.2
1	2h	P24	44h	HPC	1059	1733	1693	1793	1476	1544
2			48h		1491	1675	1590	1558	1700	1857
3			64h		1398	1368	1314	1227	1469	1761
4	1h	P21	50h		1583	1165	1479	1118	1430	1150
5					1234	1119	1234	1403	1440	1492
6					1786	1507	1827	2018	2035	1533

GMT
9.00
9.08
9.31
8.56
9.53
9.03
144.79
207.44
159.33
143.79
164.98
190.93
485.01
538.02
492.45
371.47
353.59
630.65
1527.37
1641.04
1413.52
1308.30
1313.53
1771.99
