# Supplementary material for: Methodological Validation and Inter-Laboratory Comparison of Microneutralization Assay for Detecting Anti-AAV9 Neutralizing Antibody in Human
Source: Viruses. 2024 Sep 24;16(10):1512. doi: 10.3390/v16101512 (PMC11512302; doi:10.3390/v16101512)
Supplement: Supplementary file 1 [file viruses-16-01512-s001.zip › Table S14 stability.pdf]

Table S14 stability

data on method validation in each laboratory

| Lab 1 | | | | IC50 | | | | | | analysis | | | Bias(%) | | |
|---|---|---|---|---|---|---|---|---|---|---|---|---|---|---|---|
| AR | condition | | parallel P( | test 1 | $R^2$ | test 2 | $R^2$ | test 3 | $R^2$ | test 1 | test 2 | test 3 | test 1 | test 2 | test 3 |
| 1 | basline | STB-LPC | 117 | 119 | 0.98 | 86 | 0.98 | 94 | 0.98 | 119 | 86 | 94 | | | |
| | | STB-HPC | 833 | 751 | 0.99 | 708 | 1 | 928 | 0.97 | 751 | 708 | 928 | | | |
| 2 | RT24h | STB-LPC | 101 | 60 | 0.81 | 74 | 0.97 | 123 | 0.93 | 60 | 74 | 123 | -49.6 | -14.0 | 30.9 |
| | | STB-HPC | 926 | 1681 | 0.92 | 1053 | 0.96 | 1124 | 0.98 | 1681 | 1053 | 1124 | 123.8 | 48.7 | 21.1 |
| 3 | l at -15--30℃ for 7( | STB-LPC | 53 | 58 | 0.93 | 50 | 0.82 | 76 | 0.89 | 58 | 50 | 76 | -51.3 | -41.9 | -19.1 |
| | | STB-HPC | 844 | 652 | 0.97 | 608 | 0.97 | 663 | 0.95 | 652 | 608 | 663 | -13.2 | -14.1 | -28.6 |
| 4 | l at -60--90 ℃for 7( | STB-LPC | 75 | NR | 0.79 | 115 | 0.97 | 76 | 0.96 | NR | 115 | 76 | NA | 33.7 | -19.1 |
| | | STB-HPC | 1647 | 569 | 0.98 | 666 | 0.98 | 736 | 0.99 | 569 | 666 | 736 | -24.2 | -5.9 | -20.7 |
| 5 | l at -60--90 ℃for 9' | STB-LPC | 63 | NR | 0.73 | NR | 0.78 | 54 | 0.91 | NR | NR | 54 | NA | NA | -42.6 |
| | | STB-HPC | 958 | 1076 | 0.98 | 953 | 0.98 | 1174 | 0.97 | 1076 | 953 | 1174 | 43.3 | 34.6 | 26.5 |
| 6 | 0℃ frozen-thaw for | STB-LPC | 73 | 70 | 0.98 | 58 | 0.98 | 84 | 0.95 | 70 | 58 | 84 | -41.2 | -32.6 | -10.6 |
| | | STB-HPC | 831 | 765 | 0.96 | 600 | 0.99 | 966 | 0.97 | 765 | 600 | 966 | 1.9 | -15.3 | 4.1 |
| 7 | )℃ frozen-thaw for | STB-LPC | 61 | 51 | 0.97 | 50 | 0.98 | 56 | 0.95 | 51 | 50 | 56 | -57.1 | -41.9 | -40.4 |
| 8 | l at -15--30℃ for 21 | STB-LPC | 109 | 103 | 0.96 | 87 | 0.91 | 91 | 0.93 | 103 | 87 | 91 | -13.4 | 1.2 | -3.2 |
| | | STB-HPC | 1218 | 1634 | 0.96 | 1644 | 0.96 | 990 | 0.96 | 1634 | 1644 | 990 | 117.6 | 132.2 | 6.7 |

| Lab 3 | | | | IC50 | | | | | | analysis | | | Bias(%) | | |
|---|---|---|---|---|---|---|---|---|---|---|---|---|---|---|---|
| c | condition | | | test 1 | $R^2$ | test 2 | $R^2$ | test 3 | $R^2$ | test 1 | test 2 | test 3 | test 1 | test 2 | test 3 |
| 1 | basline（RT24 ） | STB-LPC | | 119 | 0.97 | | | | | 119 | | | | | |
| | | STB-HPC | | 1269 | 0.95 | | | | | 1269 | | | | | |
| 2 | RT24h | STB-LPC | | 137 | 0.96 | 153 | 0.96 | 161 | 0.95 | 137 | 153.4 | 161 | 15.7 | 29.1 | 35.5 |
| | | STB-HPC | | 912 | 0.95 | 1122 | 0.90 | 1007 | 0.94 | 912 | 1122 | 1007 | -28.2 | -11.6 | -20.6 |
| 3 | basline（-80℃ frozen-thaw for 6 | STB-LPC | | 165 | 0.92 | | | | | 165 | | | | | |
| | | STB-HPC | | 1780 | 0.92 | | | | | 1780 | | | | | |
| 4 | -80°C frozen-thaw for 6 times | STB-LPC | | 223 | 0.95 | 211 | 0.92 | 241 | 0.95 | 223 | 210.8 | 240.8 | 35.4 | 128.1 | 46.4 |
| | | STB-HPC | | 1761 | 0.97 | 2714 | 0.92 | 1423 | 0.91 | 1761 | 2714 | 1423 | -1.1 | 52.5 | -20.1 |
| 5 | basline（stored at -80°C for | STB-LPC | | 126 | 0.97 | | | | | 126 | | | | | |
| | | STB-HPC | | 1506 | 0.88 | | | | | 1506 | | | | | |
| 6 | l at -80°C for 5mont | STB-LPC | | 155 | 0.96 | 174 | 0.99 | 197 | 0.90 | 155 | 173.7 | 196.6 | 23.4 | 38.2 | 56.4 |
| | | STB-HPC | | 1197 | 0.98 | 1181 | 0.97 | 1235 | 0.95 | 1197 | 1181 | 1235 | -20.5 | -21.6 | -18.0 |
| 7 | basline（stored at -30°C for 14 | STB-LPC | | 180 | 0.90 | | | | | 180 | | | | | |
| | | STB-HPC | | 1694 | 0.96 | | | | | 1694 | | | | | |
| 8 | red at -30°C for 14 ( | STB-LPC | | 252 | 0.94 | 172 | 0.97 | 168 | 0.97 | 252 | 172.4 | 168.3 | 40.3 | -4.0 | -6.3 |
| | | STB-HPC | | 2033 | 0.95 | 1252 | 0.97 | 1350 | 0.84 | 2033 | 1252 | 1350 | 20.0 | -26.1 | -20.3 |
