# Supplementary material for: Methodological Validation and Inter-Laboratory Comparison of Microneutralization Assay for Detecting Anti-AAV9 Neutralizing Antibody in Human
Source: Viruses. 2024 Sep 24;16(10):1512. doi: 10.3390/v16101512 (PMC11512302; doi:10.3390/v16101512)
Supplement: Supplementary file 1 [file viruses-16-01512-s001.zip › Table S15 system suitability.pdf]

Table S15 system suitability

data on method validation in each laboratory

Lab 1

	IC_{50} of LPC and HPC					GCV%	old-chang	GMT	MT rang	GMT range
LPC	NR	41	64	50	51	37	3	67	34	135
	57	93	66	NR	40					
	69	53	40	NR	63					
	97	125	72	126	53					
	NR	87	NR	47	63					
	78	85	84	82	74	GCV%	old-change			
HPC	362	639	1015	977	1038	33	3	766	383	1532
	547	768	1043	NR	547					
	789	754	451	529	332					
	1070	985	1043	1382	875					
	731	731	688	549	921					
	705	774	694	861	842					

Lab 2

	IC_{50} of LPC and HPC			GCV%	old-chang	GMT	MT rang	GMT range
LPC	61	32	52	41	3	70	35	139
	94	90	107					
	36	38	57					
	104	87	101					
	82	100	82					
HPC	389	548	477	39	3	566	283	1133
	878	716	769					
	386	492	539					
	423	442	472					
	644	626	1106					

Lab 3

**Detection content**	**Run**	**LPC**		**HPC**	
		**IC_{50}**	**R^2**	**IC_{50}**	**R^2**
Specificity	1	105	0.96	1140	1.00
-1, Drug	2	119	0.97	1269	0.95
Specificity	3	134	0.95	1672	0.96
-2 and 3	4	176	0.98	1444	0.95
Stability	5	180	0.90	1694	0.96
（RT, -80	6	143	0.97	1037	0.93
Selectivity	7	125	0.95	1466	0.98
（Normal	8	214	0.88	2269	0.85
&	9	324	0.98	955	0.99
Haemolysi	10			1594	0.96
Stability	11	229.3*	0.79	2276*	0.77
（-30 ℃	12	171	0.95	1088	0.82
Sensitivity	13	122	0.97	1361	0.95
-1	14	97	0.99	653	0.98
Sensitivity	15	172	0.94	1070	0.97
-2	16	104	0.99	813	0.98
	GMT	148		1243	
	GMT range	74		621	
	GMT range	295		2486	
	GCV%	44		16	
	Fold-change	2		2	

* denotes not meet the acceptance crieteria
