# Supplementary material for: Methodological Validation and Inter-Laboratory Comparison of Microneutralization Assay for Detecting Anti-AAV9 Neutralizing Antibody in Human
Source: Viruses. 2024 Sep 24;16(10):1512. doi: 10.3390/v16101512 (PMC11512302; doi:10.3390/v16101512)
Supplement: Supplementary file 1 [file viruses-16-01512-s001.zip › Table S16 results-inter-assay.pdf]

Table S16 results-inter-assay

IC50 of tested samples submitted by each laboratory

Lab1

sample ID	test1		test2		test3	
	IC50	R^2	IC50	R^2	IC50	R^2
S001	24380	0.95	53000	0.92	48890	0.95
S002	712	0.99	1862	0.99	1728	0.98
S003	5206	0.96	5563	0.98	5575	0.98
S004	46225	0.96	35705	0.97	32551	0.90
S005	1230	0.97	881	0.95	996	0.92
S006	1304	0.94	641	0.95	637	0.92
S007	4004	0.95	4527	0.96	3115	0.98
S008	10	-0.32	10	0.50	10	-0.39

Lab 2

sample ID	test1		test2		test3	
	IC50	R^2	IC50	R^2	IC50	R^2
S001	48340	0.9947	42517	0.9852	49440	0.9843
S002	518	0.9478	782	0.971	594	0.9898
S003	2629	0.9971	4027	0.9588	2899	0.9935
S004	64935	0.9949	56593	0.9645	47625	0.9885
S005	1062	0.9885	1346	0.993	1290	0.9761
S006	1183	0.9665	735	0.9986	702	0.8809
S007	2629	0.9838	3993	0.9878	2211	0.9892
S008	10	0.7223	10	NA	10	0.2031

Lab 3

sample ID	test1		test2		test3	
	IC50	R^2	IC50	R^2	IC50	R^2
S001	51143	0.99	82568	0.93	57703	0.93
S002	1331	0.85	1048	0.98	969.3	0.95
S003	4444	0.98	5846	0.95	5896	0.97
S004	60598	0.94	73860	0.98	82183	0.99
S005	1640	0.96	1269	0.99	1020	0.97
S006	1086	1	954.2	0.99	814.5	0.99
S007	3895	0.99	4729	0.95	5788	0.95
S008	10	NA	10	NA	10	NA

analysis data(absoulte value)

Lab1

sample ID	test1	test2	test3		Inter-assay varability(n		
	IC50	IC50	IC50	verall GM	GCV%	erall GM	Foldchange
S001	24380	53000	48890	44798			
S004(repeat of S001)	46225	35705	32551	55305	19	49775	1
S002	712	1862	1728	846			
S006(repeat of S002)	1304	641	637	655	59	744	1
S003	5206	5563	5575	3233			
S007(repeat of S003)	4004	4527	3115	3167	24	3200	2
S005	1230	881	996	1154	19	1026	1
S008	10	10	10	10	0	10	1

Lab 2

sample ID	test1	test2	test3		Inter-assay varability(n		
	IC50	IC50	IC50	verall GM	GCV%	erall GM	Foldchange
S001	48340	42517	49440	44798			
S004(repeat of S001)	64935	56593	47625	55305	19	49775	1
S002	518	782	594	846			
S006(repeat of S002)	1183	735	702	655	35	744	1
S003	2629	4027	2899	3233			
S007(repeat of S003)	2629	3993	2211	3167	24	3200	1
S005	1062	1346	1290	1154	13	1226	1
S008	10	10	10	10	0	10	1

Lab 3

sample ID	test1	test2	test3		Inter-assay varability(n		
	IC50	IC50	IC50	verall GM	GCV%	erall GM	Foldchange
S001	51143	82568	57703	62460			
S004(repeat of S001)	60598	73860	82183	71650	21	#####	2
S002	1331	1048	969	1106			
S006(repeat of S002)	1086	954	815	945	18	1022	1
S003	4444	5846	5896	5351			
S007(repeat of S003)	3895	4729	5788	4742	18	5037	2
S005	1640	1269	1020	1285	26	1285	2
S008	10	10	10	10	0	10	1
