# Supplementary material for: Methodological Validation and Inter-Laboratory Comparison of Microneutralization Assay for Detecting Anti-AAV9 Neutralizing Antibody in Human
Source: Viruses. 2024 Sep 24;16(10):1512. doi: 10.3390/v16101512 (PMC11512302; doi:10.3390/v16101512)
Supplement: Supplementary file 1 [file viruses-16-01512-s001.zip › Table S17 results-inter-intra-lab.pdf]

Table S17 results-inter-intra-lab

	IC_{50}						Intra-lab variability			Inter-lab variability		
							GMT	%GCV	foldchange	GMT	%GCV	foldchange
	24380	46225	53000	35705	48890	32551	38766	31	2			
S001/S004	48340	64935	42517	56593	49440	47625	51090	17	1	50980	33	3
	51143	60598	82568	73860	57703	82183	66897	21	1			
	712	1304	1862	641	1728	637	1034	59	3			
S002/S006	518	1183	782	735	594	702	726	35	2	915	46	4
	1331	1086	1048	954	969	815	1022	18	1			
	5206	4004	5563	4527	5575	3115	4571	23	2			
S003/S007	2629	2629	4027	3993	2899	2211	2988	28	2	4098	32	2
	4444	3895	5846	4729	5896	5788	5037	18	1			
	1230	881	996				1026	19	1			
S005-IVIG	1346	885	1290				1154	24	2	1150	23	2
	1640	1269	1020				1285	26	2			
	10	10	10				10	0	1			
S008	10	10	10				10	0	1	0	0	1
	10	10	10				10	0	1			

	cut-off titer						Intra-lab variability			Inter-lab variability		
							GMT	%GCV	foldchange	GMT	%GCV	foldchange
	20480	40960	40960	20480	20480	20480	25803	45				
S001/S004	40960	40960	40960	40960	40960	81920	45976	40		36491	42	
	40960	40960	40960	40960	40960	40960	40960	0				
	640	1280	1280	640	320	320	640	72				
S002/S006	320	320	320	640	320	640	403	45		593	97	
	640	640	2560	640	640	640	806	104				
	2560	5120	5120	2560	2560	2560	3225	45				
S003/S007	2560	2560	1280	2560	1280	2560	2032	35		2765	49	
	2560	5120	5120	2560	2560	2560	3225	45				
	640	640	640				640	0				
S005-IVIG	1280	640	640				806	51		806	44	
	1280	640	1280				1016	40				
	10	10	10				10	0				
S008	10	10	10				10	0		10	0	
	10	10	10				10	0				
