# Supplementary material for: Methodological Validation and Inter-Laboratory Comparison of Microneutralization Assay for Detecting Anti-AAV9 Neutralizing Antibody in Human
Source: Viruses. 2024 Sep 24;16(10):1512. doi: 10.3390/v16101512 (PMC11512302; doi:10.3390/v16101512)
Supplement: Supplementary file 1 [file viruses-16-01512-s001.zip › Table S2. Study sample set.pdf]

Table S2. Study sample set	
Sample label	Description
S001	human serum collecting on Day 360 after rAAV9-hCoGAA (GC301) treatment via *i.v.*
S002	human EDTA K_2-anticoagulated plasma collecting on Day 56 after rAAV9-hSMN1 (GC101) treatment via *i.t.*
S003	human serum collecting on Day 14 after rAAV9-hSMN1 (GC101) treatment via i.t.
S004	Repeat of S001
S005	Commercial human IVIG-purified immunoglobulin
S006	Repeat of S002
S007	Repeat of S003
S008	Pooled human negative sera

The delivery route for rAAV9-based drugs, GC101 and GC301 is intrathecal infusion (*i.t*) and intravenous infusion (*i.v*), respectively.
