# Supplementary material for: Methodological Validation and Inter-Laboratory Comparison of Microneutralization Assay for Detecting Anti-AAV9 Neutralizing Antibody in Human
Source: Viruses. 2024 Sep 24;16(10):1512. doi: 10.3390/v16101512 (PMC11512302; doi:10.3390/v16101512)
Supplement: Supplementary file 1 [file viruses-16-01512-s001.zip › Table S3 List of Abbreviations.pdf]

List of Abbreviations	
S	sample
AR	analytical run
PC	positive control
LPC	low-quality positive control
FT	frozen/thaw
MPC	moderate-quality positive control
HPC	high-quality positive control
SPC	specificity
SEL	selectivity
STB	stability
LTS	long term stability
Lip	lipidemia
NC	negative quality control
NR	not report
NA	not available
Con.	Concentration
