# Supplementary material for: Methodological Validation and Inter-Laboratory Comparison of Microneutralization Assay for Detecting Anti-AAV9 Neutralizing Antibody in Human
Source: Viruses. 2024 Sep 24;16(10):1512. doi: 10.3390/v16101512 (PMC11512302; doi:10.3390/v16101512)
Supplement: Supplementary file 1 [file viruses-16-01512-s001.zip › Table S4 TCP.pdf]

Table S4 TCP
data on method validation in each laboratory

Lab 1 TCP=21

Sample ID	IC_{50}					
	Day 1	Day 2	Day 3	Day 4	Day 5	Day 6
S 1	10	18	23	NA	10	10
S 2	10	17	NA	10	10	15
S 3	10	14	19	10	10	19
S 4	10	10	10	10	10	10
S 5	10	23	NA	10	10	10
S 6	10	10	28	20	8	10
S 7	10	14	NA	10	10	19
S 8	12	10	11	10	10	10
S 9	10	10	10	NA	10	10
S 10	10	10	19	20	24	10
S 11	20	10	10	10	20	10
S 12	21	16	10	10	17	10
S 13	9	10	10	19	10	10
S 14	10	11	10	10	10	10
S 15	10	10	10	10	15	10
S 16	10	10	10	10	10	NA
S 17	10	10	NA	10	NA	10
S 18	10	10	10	10	10	10
S 19	10	10	10	10	10	10
S 20	18	21	20	10	21	10
S 21	19	19	15	5	10	10
S 22	10	10	10	10	10	10
S 23	22	10	10	10	10	10
S 24	10	10	10	10	10	20
S 25	20	20	10	10	10	30
S 26	10	10	24	10	10	10

Lab 3 TCP=15

Sample ID	IC_{50}					
	Day 1	Day 2	Day 3	Day 4	Day 5	Day 6
1	10	10	10	10	10	10
2	10	10	43	10	10	10
3	10	10	10	10	10	10
4	10	10	10	10	10	10
5	10	10	10	10	10	10
6	10	10	10	10	10	10
7	10	10	10	10	10	10
8	26	10	10	10	10	10
9	10	10	10	10	10	10
10	10	10	10	10	10	10
11	10	10	10	10	10	10
12	10	10	10	10	10	10
13	16	10	10	10	10	10
14	10	10	10	10	10	10
15	47	10	10	10	25	18
16	10	10	10	10	10	10
17	10	10	10	10	10	10
18	10	10	10	10	10	10
19	10	10	10	10	10	10
20	10	10	10	10	10	10
21	10	44	10	10	10	22
22	10	10	10	10	10	10
23	10	10	10	10	10	19
24	10	10	10	10	10	10
25	10	10	10	8	10	10
26	10	10	10	10	10	10
27	10	10	19	15	8	9
28	10	10	10	14	10	11
29	10	10	10	1	10	5
30	10	10	10	10	10	3
31	10	10	10	10	10	1
32	10	10	10	10	10	10
33	10	10	10	10	10	10
34	10	10	10	4	10	10
35	10	10	10	10	10	3
36	10	10	10	4	1	10
37	10	10	10	10	10	10
38	10	10	10	10	8	10
39	10	10	10	10	10	10
40	10	10	10	1	10	2
41	10	10	10	10	10	10
42	10	10	10	3	10	10
43	10	10	10	NA	10	10
44	10	10	10	3	3	10
45	10	10	10	11	10	10
46	10	10	10	10	10	10
47	10	10	10	10	10	10
48	10	10	10	10	10	10
49	10	10	10	18	10	10
50	10	10	10	10	10	10
51	10	10	10	14	10	10

NA denotes the outliers from the raw data using the Tukey box-plot outlier
