# Supplementary material for: Methodological Validation and Inter-Laboratory Comparison of Microneutralization Assay for Detecting Anti-AAV9 Neutralizing Antibody in Human
Source: Viruses. 2024 Sep 24;16(10):1512. doi: 10.3390/v16101512 (PMC11512302; doi:10.3390/v16101512)
Supplement: Supplementary file 1 [file viruses-16-01512-s001.zip › Table S5 precision lab1.pdf]

Table S5 precision lab1

data on method validation in each laboratory

Lab 1		IC50						Intra-assay variability [illegible]			Inter-assay variability (n=36)		
		AR1	AR2	AR3	AR4	AR5	AR6	GCV%	Overall IC_{50} GMT	Fold change	GCV%	Overall IC_{50} GMT	Fold change
NC	Day1	10	10	10	10	10	10	0	10	1	39	11	3
	Day2	23	10	24	10	10	10	58	13	2			
	Day3	20	10	10	10	10	10	40	11	1			
	Day4	20	14	18	10	9	10	40	13	2			
	Day5	10	15	10	10	10	10	21	11	2			
	Day6	13	10	10	10	10	12	13	11	1			
		AR1	AR2	AR3	AR4	AR5	AR6	GCV%	Overall IC_{50} GMT	Fold change	GCV%	Overall IC50 GMT	Fold change
LPC	Day1	NR	41	64	50	51	67	22	54	2	37	68	3
	Day2	57	93	66	NR	40	71	34	63	2			
	Day3	69	53	40	NR	63	50	23	54	2			
	Day4	97	125	72	126	53	110	35	93	2			
	Day5	NR	87	NR	47	63	65	28	64	2			
	Day6	78	85	84	82	74	90	7	82	1			
		AR1	AR2	AR3	AR4	AR5	AR6	GCV%	Overall IC_{50} GMT	Fold change	GCV%	Overall IC50 GMT	Fold change
MPC	Day1	102	138	204	172	156	141	25	149	2	30	161	3
	Day2	116	151	117	145	98	188	26	133	2			
	Day3	137	NR	83	115	NR	178	36	124	2			
	Day4	277	200	185	235	171	206	20	210	1			
	Day5	164	140	112	136	180	194	22	152	2			
	Day6	188	231	197	201	205	234	9	209	1			
		AR1	AR2	AR3	AR4	AR5	AR6	GCV%	Overall IC_{50} GMT	Fold change	GCV%	Overall IC50 GMT	Fold change
HPC	Day1	362	639	1015	977	1038	1155	52	806	3	33	766	4
	Day2	547	768	1043	NR	547	NR	50	700	2			
	Day3	789	754	451	529	332	779	50	576	2			
	Day4	1070	985	1043	1382	875	1023	43	1053	2			
	Day5	731	731	688	549	921	747	43	720	2			
	Day6	705	774	694	861	842	921	41	795	1			
