# Supplementary material for: Methodological Validation and Inter-Laboratory Comparison of Microneutralization Assay for Detecting Anti-AAV9 Neutralizing Antibody in Human
Source: Viruses. 2024 Sep 24;16(10):1512. doi: 10.3390/v16101512 (PMC11512302; doi:10.3390/v16101512)
Supplement: Supplementary file 1 [file viruses-16-01512-s001.zip › Table S6 precision lab2.pdf]

Table S6 precision lab2

data on method validation in each laboratory

Lab 2		IC50			Intra-assay variability (n=3)			Inter-assay variability (n=15)		
		AR1	AR2	AR3	GCV%	Overall IC_{50} GMT	Fold change	GCV%	Overall IC50 GMT	Fold change
NC	Day1	10	10	10	0	10	1	19	10	2
	Day2	10	10	17	38	12	2			
	Day3	10	10	10	0	10	1			
	Day4	10	10	10	0	10	1			
	Day5	10	10	10	0	10	1			
		AR1	AR2	AR3	GCV%	Overall IC50 GMT	Fold change	GCV%	Overall IC50 GMT	Fold change
LPC	Day1	61	32	52	35	47	2	41	70	3
	Day2	94	90	107	10	97	1			
	Day3	36	38	57	30	43	2			
	Day4	104	87	101	10	97	1			
	Day5	82	100	82	12	88	1			
		AR1	AR2	AR3	GCV%	Overall IC50 GMT	Fold change	GCV%	Overall IC50 GMT	Fold change
MPC	Day1	121	109	149	18	125	1	28	160	2
	Day2	191	111	144	30	145	2			
	Day3	105	132	202	39	141	2			
	Day4	215	211	182	9	202	1			
	Day5	209	203	194	4	202	1			
		AR1	AR2	AR3	GCV%	Overall IC50 GMT	Fold change	GCV%	Overall IC50 GMT	Fold change
HPC	Day1	389	548	477	18	467	1	39	566	3
	Day3	878	716	769	11	785	1			
	Day4	386	492	539	18	468	1			
	Day5	423	442	472	6	445	1			
	Day6	644	626	1106	40	764	2			
