# Supplementary material for: Methodological Validation and Inter-Laboratory Comparison of Microneutralization Assay for Detecting Anti-AAV9 Neutralizing Antibody in Human
Source: Viruses. 2024 Sep 24;16(10):1512. doi: 10.3390/v16101512 (PMC11512302; doi:10.3390/v16101512)
Supplement: Supplementary file 1 [file viruses-16-01512-s001.zip › Table S7 precision lab3.pdf]

Table S7 precision lab3

data on method validation in each laboratory

Lab 3		IC50						Intra-assay variability (n=3)			Inter-assay variability (n=36)		
		AR1	AR2	AR3	AR4	AR5	AR6	GCV%	Overall IC_{50} GMT	Fold change	GCV%	Overall IC_{50} GMT	Fold change
NC	Day1	10	8	7	10	10	10	15	9	1	19	9	2
	Day2	10	6	10	10	10	10	21	9	2			
	Day3	6	10	10	10	10	10	16	9	2			
	Day4	10	4	10	10	10	10	31	9	3			
	Day5	8	10	10	10	10	10	11	10	1			
	Day6	10	10	10	10	5	10	22	9	2			
		AR1	AR2	AR3	AR4	AR5	AR6	GCV%	Overall IC_{50} GMT	Fold change	GCV%	Overall IC_{50} GMT	Fold change
LPC	Day1	112	189	130	158	137	155	20	145	2	22	167	2
	Day2	201	173	239	231	220	189	13	207	1			
	Day3	136	164	164	107	213	195	26	159	2			
	Day4	157	111	156	130	145	174	17	144	2			
	Day5	154	137	162	220	153	176	19	165	2			
	Day6	191	164	204	203	199	188	8	191	1			
		AR1	AR2	AR3	AR4	AR5	AR6	GCV%	Overall IC_{50} GMT	Fold change	GCV%	Overall IC_{50} GMT	Fold change
MPC	Day1	370	638	535	505	465	440	20	485	2	29	469	2
	Day2	369	491	594	551	636	645	21	538	2			
	Day3	524	515	466	352	491	657	21	492	2			
	Day4	333	361	516	322	434	304	24	371	2			
	Day5	268	304	414	469	343	361	22	354	2			
	Day6	624	609	767	607	570	624	11	631	1			
		AR1	AR2	AR3	AR4	AR5	AR6	GCV%	Overall IC_{50} GMT	Fold change	GCV%	Overall IC_{50} GMT	Fold change
HPC	Day1	1059	1733	1693	1793	1476	1544	19	1527	2	18	1486	2
	Day2	1491	1675	1590	1558	1700	1857	8	1641	1			
	Day3	1398	1368	1314	1227	1469	1761	14	1414	1			
	Day4	1583	1165	1479	1118	1430	1150	16	1308	1			
	Day5	1234	1119	1234	1403	1440	1492	12	1314	1			
	Day6	1786	1507	1827	2018	2035	1533	14	1772	1			
