# Supplementary material for: Methodological Validation and Inter-Laboratory Comparison of Microneutralization Assay for Detecting Anti-AAV9 Neutralizing Antibody in Human
Source: Viruses. 2024 Sep 24;16(10):1512. doi: 10.3390/v16101512 (PMC11512302; doi:10.3390/v16101512)
Supplement: Supplementary file 1 [file viruses-16-01512-s001.zip › Table S8 sensitivity and hook effects.pdf]

Table S8 sensitivity and hook effects

data on method validation in each laboratory							analysis data
Lab 1							
	IC$_{50}$						
concentration of anti-AAV9 MoAb(ng/Ml)	Day 1	Day 2	Day 3	Day 4	Day 5	Day 6	
3200	1344	1132	2398	1346	1972	2444	
1600	642	610	1235	622	1030	1167	
800	229	254	551	407	504	486	
400	92	118	541	151	247	371	
200	82	93	98	121	100	85	
100	39	58	49	73	38	38	
50	25	28	37	34	22	10	
25	14	16	21	22	10	10	mean
caculated PC concentration in neat plasma (ng/mL)	50	50	50	25	50	100	54
Lab 2							
concentration of anti-AAV9cMoAb(ng/mL)	Day 1	Day 2	Day 3	Day 4	Day 5	Day 6	
200	75	70	82	79	100	116	
100	57	37	60	48	38	/	
50	36	26	29	25	28	45	
25	23	24	/	19	10	10	mean
caculated PC concentration in neat plasma (ng/mL)	50	25	NR	50	50	50	45

Lab 3

hook

QC	Con. (ng/mL)	Day 1	Day 2	Day 3	Day 4	Day 5
LPC	100	228	127	122	166	100
MPC	500	932	708	1090	557	450
HPC	10000	12990	6403	9794	6360	6328

sensitivity

concentration of anti-AAV9 MoAb(ng/mL)	Day 1	Day 2	Day 3	Day 4	Day 5	
100ng	87	95	54	113	64	
50ng	55	34	56	30	28	
25ng	10	27	22	29	22	
10ng	17	12	10	10	10	
5ng	10	28	10	11	9	mean
caculated PC concentration in neat	10	10	25	5	25	15

NR is the QCs do not meet the acceptance criteria.
