# Supplementary material for: Methodological Validation and Inter-Laboratory Comparison of Microneutralization Assay for Detecting Anti-AAV9 Neutralizing Antibody in Human
Source: Viruses. 2024 Sep 24;16(10):1512. doi: 10.3390/v16101512 (PMC11512302; doi:10.3390/v16101512)
Supplement: Supplementary file 1 [file viruses-16-01512-s001.zip › Table S9 drug tolerance.pdf]

Table S9 drug tolerance

QC	Con.	with GC301 10^9/ml		with GC301 10^8/ml		with GC301 10^7/ml		Parallel PC	
	ng/mL	IC_{50}	R^2	IC_{50}	R^2	IC_{50}	R^2	IC_{50}	R^2
HPC	2000	891	0.98	1057	0.97	1035	0.97	1269	0.95
LPC	200	162	0.96	146	0.91	189	0.92	119	0.97
NC	0	10	NA	10	NA	10	NA	10	NA
